# Potential of Genome-Wide Studies in Unrelated Plus Trees of a Coniferous Species, *Cryptomeria japonica* (Japanese Cedar)

**DOI:** 10.3389/fpls.2018.01322

**Published:** 2018-09-10

**Authors:** Yuichiro Hiraoka, Eitaro Fukatsu, Kentaro Mishima, Tomonori Hirao, Kosuke M. Teshima, Miho Tamura, Miyoko Tsubomura, Taiichi Iki, Manabu Kurita, Makoto Takahashi, Atsushi Watanabe

**Affiliations:** ^1^Forest Tree Breeding Center, Forestry and Forest Products Research Institute, Hitachi, Japan; ^2^Kyushu Regional Breeding Office, Forest Tree Breeding Center, Forestry and Forest Products Research Institute, Kumamoto, Japan; ^3^Forest Bio-Research Center, Forestry and Forest Products Research Institute, Hitachi, Japan; ^4^Faculty of Science, Kyushu University, Fukuoka, Japan; ^5^Faculty of Agriculture, Kyushu University, Fukuoka, Japan; ^6^Tohoku Regional Breeding Office, Forest Tree Breeding Center, Forestry and Forest Products Research Institute, Takizawa, Japan

**Keywords:** *Cryptomeria japonica*, first-generation plus trees, genomic prediction, genome-wide association study (GWAS), linkage disequilibrium, population structure, unrelated genotypes

## Abstract

A genome-wide association study (GWAS) was conducted on more than 30,000 single nucleotide polymorphisms (SNPs) in unrelated first-generation plus tree genotypes from three populations of Japanese cedar *Cryptomeria japonica* D. Don with genomic prediction for traits of growth, wood properties and male fecundity. Among the assessed populations, genetic characteristics including the extent of linkage disequilibrium (LD) and genetic structure differed and these differences are considered to be due to differences in genetic background. Through population-independent GWAS, several significant SNPs found close to the regions associated with each of these traits and shared in common across the populations were identified. The accuracies of genomic predictions were dependent on the traits and populations and reflected the genetic architecture of traits and genetic characteristics. Prediction accuracies using SNPs selected based on GWAS results were similar to those using all SNPs for several combinations of traits and populations. We discussed the application of genome-wide studies for *C. japonica* improvement.

## Introduction

Elucidating genetic control of various objective traits of forest trees enables increased economic efficiency of forestry, improved quality of forest products and provides direction for meeting societal expectations regarding environmental issues. Due to the long time to maturation for forest tree species, evaluation of traits and breeding and phenotyping strategies are cost- and time-prohibitive, while genome-wide studies including genome-wide association study (GWAS) and genomic selection (GS) strategies are innovative, attractive and effective methodologies (Grattapaglia and Resende, 2011; Iwata et al., 2011; Uchiyama et al., 2013). With the development of technologies for high-throughput sequencing and genotyping for markers such as single nucleotide polymorphisms (SNPs), these methodologies are becoming possible. GWAS enables detection of quantitative trait loci (QTL) or causal genes from the association between genome-wide markers and phenotypes of target traits and outperforms bi-parental QTL mapping because GWAS does not require the development of segregating populations. GS predicts individual genetic merit using a large number of DNA markers such as SNPs (Meuwissen et al., 2001). GS promises to significantly reduce the time needed to achieve animal and crop improvement by skipping the time- and labor-consuming field testing stages and thus to increase genetic gain per unit time (Burdon and Wilcox, 2011; Grattapaglia and Resende, 2011).

For rapid improvement of various objective traits that impact social needs, it is effective to construct genome-wide studies based on populations consisting of genetically diverse or unrelated individuals. Predicting breeding values of unrelated individuals is exactly required in the most promising applications of GS (Meuwissen, 2009). However, particularly in conifers, genome-wide studies are more difficult compared to other animals and crops because of the large genome sizes (Chagné et al., 2003; Neale et al., 2014) and low linkage disequilibrium (LD) due to being undomesticated (Neale and Savolainen, 2004; Uchiyama et al., 2013; Neale et al., 2014; Plomion et al., 2014; Isik et al., 2016). GS accuracies in coniferous species dropped to very low when predictions were conducted with completely unrelated progeny and families (Beaulieu et al., 2014a,b; Lenz et al., 2017). Indeed, almost all previous GS studies in coniferous species examined populations consisting of related individuals such as full- or half-sibs (Resende et al., 2012a,b,c; Zapata-Valenzuela et al., 2012; Beaulieu et al., 2014a,b; De La Torre et al., 2015; Ratcliffe et al., 2015; Bartholomé et al., 2016; Isik et al., 2016; Lenz et al., 2017). For the success of GWAS and GS in populations consisting of unrelated individuals, population genetics, e.g., population structure and intensity of the LD, are important aspects. The genetic background is affected by population histories such as population demography, domestication history and selection schemes (Dunning et al., 2000; Meadows et al., 2008; Slatkin, 2008; Gray et al., 2009; Rossi et al., 2009; Carneiro et al., 2011; Hamblin et al., 2011; Akagi et al., 2016; Campoy et al., 2016). Thus, population structures need to be evaluated prior to implementation of GWAS and GS.

A coniferous species, Japanese cedar (*Cryptomeria japonica* D. Don), which is a member of the Cupressaceae botanical family, is an endemic species to Japan and is one of the most important forestry species in the country, accounting for 44% of the plantation area in Japan. The improvement program for the species was started in 1957, and 3,670 phenotypically superior first-generation plus trees were selected from artificial and natural forests throughout Japan and were clonally conserved (Forest and Forestry Products Research Institute, Forest Tree Breeding Center, 2016). Phenotypic traits, such as growth and wood quality, have been intensively evaluated for a large proportion of the plus trees in more than 1,300 trials, including in clonal and progeny test sites throughout Japan. The plus trees had been selected from across most of the distribution area throughout the long archipelago, which varies greatly in climate and, thus, the plus tree populations are expected to have adaptions to diverse environments (Miyamoto et al., 2014). Furthermore, the plus trees possess genetic diversity that is similar to that in natural populations because the core collection of individuals showed genetic diversity comparable to or higher than that of natural populations (Uchiyama et al., 2014). Because artificial forests were considered to be constructed using seedlings derived from nearby natural forests, the artificial forests of *C. japonica* were considered to have expanded gradually from areas adjacent to the natural forests and to have inherited genes from those natural forests, giving plus trees a shared genetic background (Tomaru, 1992; Miyamoto et al., 2014). Therefore, the plus trees of *C. japonica* are important both as source materials for further breeding and research activities and as genetic resources (Miyamoto et al., 2014). Additionally, the first-generation plus trees of *C. japonica* enable us to carry out genome-wide studies based on these diverse resources and an enormous database of phenotypic information.

In order to link phenotypes to genotypes in the genome-wide studies for coniferous species such as *C. japonica*, a very large number of markers would be required (Iwata et al., 2011; Lu et al., 2016). Recently, a genotyping platform with more than 70,000 *C. japonica* SNPs was developed by resequencing expressed sequence tags (ESTs) and genotyping platforms (Mishima et al., 2018). Based on the genotyping platforms, marker-assisted selection (MAS) for male sterility was successfully carried out by QTL analysis with an F_2_ mapping population (Mishima et al., 2018). The genotyping platforms would also allow genome-wide studies for various quantitative traits. In an empirical genome-wide study for *C. japonica*, Uchiyama et al. (2013) used GWAS for wood property traits and quantity of male strobili (male fecundity) based on *C. japonica* plus trees using 1,032 SNPs and identified several significant markers. In this study, we are able to perform genome-wide studies using a significantly larger number of SNPs than were used in previous studies.

In the present study, we reveal the potential of genome-wide studies in a coniferous species using unrelated *C. japonica* plus trees from multiple breeding populations, which would have experienced different histories and showed different genetic characteristics. Understanding genetic diversity and population structure is a necessary step in formulating strategies of genetic improvement and conservation and is urgently needed for evaluating and modifying the breeding program (Jin et al., 2016). Using the plus trees from such multiple breeding populations, we attempt (1) to examine the genetic background of the *C. japonica* plus tree populations, (2) to perform GWAS and genomic prediction for several traits, and (3) to clarify the relationship between the results of genomic prediction and the population structure. The targeted traits in this study were growth, wood properties and male fecundity. Further, we discuss future applications of genome-wide studies in *C. japonica* based on this study.

## Materials and Methods

### Plant Materials and DNA Extraction

A total of 476 plus trees from two breeding regions (Kanto and Kyushu) were selected for sampling in this study (**Supplementary Table S1**). Plus trees in the Kanto breeding region were selected from two populations, N-Kanto and S-Kanto, which are located in northern-inland and southern-coastal areas of the Kanto breeding region, respectively (**Supplementary Figure S1**). The regions from where these populations originated showed the same cluster in STRUCTURE analysis with *K* = 2 in the population genetics study based on the core collection of plus trees using 1000s of SNPs (see Figure 2 in Uchiyama et al., 2014). All plus tree genotypes belonging to the two Kanto populations were preserved in clonal archives at Forest Tree Breeding Center (FTBC) in Hitachi, Ibaraki and those from the Kyushu population at the Kyushu Regional Breeding Office of FTBC. Current fresh shoots were sampled from all sample genotypes and stored at -20°C. Total DNA was extracted from the sampled shoots using DNeasy Plant Mini Kit (QIAGEN, Hilden, Germany). These were genotyped using four simple sequence repeat markers, and the genotypes were confirmed to be different within populations (data not shown).

### Genotype Data

For SNP genotyping, we performed Affymetrix Axiom genotyping using GeneTitan^®^ system with Axiom_Cj_70K_ver. 1.0 [73,274 SNPs; Gene Expression Omnibus Dataset (GEO): GSE95616] or Axiom_Cj_70K_ver. 2.0 (73,640 SNPs; GEO: GSE95618) arrays (Mishima et al., 2018). SNP data obtained from the 53,378 SNP markers found in common on both arrays were used for the following analysis with processing as follows. First, SNPs categorized as monomorphic were removed. Then, SNPs with a missing data ratio > 50% were removed. After that, SNPs with minor allele frequency (MAF) below 5% were discarded. The resulting 32,036 markers of 476 genotypes were used for the following analysis. The SNP data were converted to the scores (–1, 0 or 1), and the missing SNP data points were imputed by the “A.mat” function with impute.method = “EM” in the “rrBLUP” package (Endelman, 2011) in R 3.3.3 (R Core Team, 2017).

### Linkage Disequilibrium and Genetic Structure

Mishima et al. (2018) constructed a linkage map of the *C. japonica* F_2_ family based on 6,629 markers including SNPs used in this study and simple sequence repeat markers. Here, we used the map information for the 6,455 SNPs that remained following our SNP selection process described above. We calculated the intensity of LD (*r*^2^) between the mapped SNPs within the same linkage group (LG). LD was calculated the “LD” function in the “genetics” package in R (Warnes et al., 2013).

We calculated the average expected (*H*_E_) and observed (*H*_O_) heterozygosities using the “basicStats” function of the “diveRsity” package in R (Keenan et al., 2013). We performed ancestry analysis for populations using the “snmf” function of the “LEA” package in R (Frichot and François, 2015) based on the mapped 6,455 SNPs (data used before being imputed). To choose the number of clusters (*K*), the cross entropy criterion that was calculated by the snmf function with “entropy = TRUE” option with *K* = 1–8, was used. In order to evaluate the magnitude of admixture in each genotype for each population, we proposed the effective number of clusters per genotype (*N*_Q_) using the following equation

$$N_{Q}=\overset{K}{\underset{i=1}{\Sigma }}\frac{1}{Q_{i}^{2}}$$

where *Q*_i_ is an individual assignment probability of the *i*th cluster within each genotype and *K* is the *K* value set in the ancestry analysis. The value of *N*_Q_ has a range of 1–*K*, and when the magnitude of the admixture is stronger, the value becomes higher. We also conducted principal component analysis (PCA) for all tested populations together by the “prcomp” function in R.

### Phenotypic Data

In this study, we assessed three important traits for genomic prediction, growth traits (height and diameter at breast height, DBH), wood properties (wood stiffness and density) and reproductive traits (male fecundity) using clonal propagated plus tree individuals.

Growth traits were evaluated at age 10 years using plot mean data obtained at clonal test sites. Almost all clonal test sites had incomplete random block design with three replications. Dozens of genotypes were planted at each test site, and there were several overlaps of genotypes between the test sites. Each replication had multiple plots, and >10 individuals of each genotype were planted within each plot. For the analysis of growth traits, we used growth data obtained from 137 and 132 clonal test sites in Kanto and Kyushu regions, respectively. Genetic values were calculated as phenotype data of growth traits for genomic prediction using a linear mixed model based on the BLUP (best linear unbiased prediction) method using ASReml 3 software (VSN International, Hemel Hempstead, United Kingdom). The following mixed linear model was used:

$$y_{ijk}=\mu +S_{i}+{S\left(B\right)}_{ij}+C_{k}+{\left(SC\right)}_{ik}+e_{ijk}$$

where *y*_ijk_ is a plot mean value of the *k*th genotype at the *j*th block within *i*th test site; *μ* is the overall mean; (*SC*)_ik_ is the interaction effect of the *i*th test site and *k*th genotype; and *e*_ijk_ is the residual. The fixed effects included *μ*, *S*_i_ and *S(B)*_ij_, and the others were random effects. The parameter *C*_k_ was assumed to be the genetic value of height or DBH.

Wood stiffness and density were evaluated by stress wave velocity measured with TreeSonic Microsecond Timer (Fakopp, Hungary) or Fakopp Microsecond Timer (Fakopp, Hungary) and by penetration depth with Pilodyn 6J Forest (Proceq, Switzerland) according to the procedure described by Mishima et al. (2011). Genotypes from N-Kanto and S-Kanto populations were assessed together in the Ohkubo stock garden of FTBC in Hitachi, Ibaraki (36°33′N, 140°36′E). Clonal replications were in the range 2–3 at the site. As the clonal value of each trait, we averaged the scores from individuals of each genotype. The data in N-Kanto and S-Kanto used the same values obtained by Mishima et al. (2011). For genotypes from the Kyushu populations, stress wave velocity was assessed in clonal archives in Koshi, Kumamoto (32°53′N, 130°44′E) and in Mifune, Kumamoto (32°47′N, 130°57′E), and wood density was assessed in these two clonal archives in Kumamoto and another 12 clonal test sites. The number of sites and ramets used for each genotype is unbalanced; therefore, the BLUP value obtained from the mixed linear model (formulae 2) was used as a clonal value for each genotype.

For evaluation of male fecundity, male strobili quantity index was measured according to the procedure described by Tsubomura et al. (2013). Briefly, after treatment of 100 ppm gibberellin (GA_3_) on three shoots in early July of the same year, the index (1: less – 5: much) for strobili quantity on each individual tree was recorded by multiple observers in December, and the average from the three shoots was taken as a measure of male fecundity for each individual. Genotypes from N-Kanto and S-Kanto populations were assessed together at a clonal garden in FTBC, and those from the Kyushu population were assessed at a clonal garden at the Kyushu Regional Breeding Office; clonal replication was in the range of 2–3 at both sites. As the clonal value, we averaged the scores from individuals of each genotype. The data of genotypes in N-Kanto and S-Kanto were the same as reported in Tsubomura et al. (2013) and obtained in Kyushu for this study.

The N-Kanto and S-Kanto populations were evaluated together for genotype traits and compared with those in the Kyushu population because individuals from N-Kanto and S-Kanto were planted together. The numbers of analyzed data and genotypes for each trait within each breeding region are shown in **Supplementary Table S2**. Based on the dataset, significance of genetic effects for the objective traits was confirmed by *F* test or a likelihood ratio test according to Self and Liang (1987), which was employed for comparison with no genetic factor models. For the likelihood ratio test, a value of deviance, Δ*D*_1,2_, was calculated using the following equation:

$$\Delta D_{1,2}=-2\times \left(\log L_{1}^{*}-\log L_{2}^{*}\right)$$

where log $L_{1}^{*}$ and log $L_{2}^{*}$ are maximum log-likelihoods of models without and with genetic factor, respectively. Δ*D*_1,2_ follows a chi-square distribution with 1 degree of freedom. The genetic effects were statistically significant for all assessed traits in this study (**Supplementary Table S2**). The genotypes used for GWAS and genomic prediction (**Supplementary Table S1**) were all included in the phenotypically evaluated genotypes in **Supplementary Table S2**.

### Procedure for GWAS

We carried out GWAS for each population and each trait using all 32,036 SNPs in the “GWAS” function of the rrBLUP package in R. In the function, the “K” option, which specifies the covariance between genotypes, was set with a kinship matrix between genotypes calculated by the “A.mat” function of the rrBLUP package and the “n.PC” option, which specifies the number of principal components (PC) to include as fixed effects, was determined by the variances of PC scores based on the PC analysis (PCA) for each population (**Supplementary Figure S2**); the values were set to 2, 6 and 5 in the N- and S-Kanto and Kyushu populations, respectively. We used a false discovery rate (FDR) < 0.1 or -log_10_(*P*) > 3 as criteria for statistically significant GWAS results. We calculated *q*-values for FDR using the “p.adjust” function in R. For estimation of unmapped SNP positions which showed -log_10_(*P*) > 3 in GWAS, we conducted LD calculation with the 6,455 mapped SNPs. If the highest *r*^2^ was more than 0.6, the unmapped SNP position was assumed as being equal to that of the paired SNP. We independently performed GWAS for each population and then detected significant SNPs at similar map positions (<10 cM) across the populations for identifying commonly significant genome regions. *Arabidopsis* homologs were used as searches for significant loci within the TAIR10 database using BLASTN with an *E*-value cutoff of 1E^-5^.

### Genomic Prediction and Validation

We performed genomic prediction for each population and each trait using three methods: genomic best linear unbiased prediction (GBLUP), BayesB and Random Forest (RF). GBLUP was performed by using the “kin.BLUP” function of the rrBLUP package of R. BayesB was performed by using the “BGLR” function of the “BGLR” package of R (Pérez and de los Campos, 2014) with a 10,000 burn-in and 20,000 iteration settings. We also performed RF with the “randomForest” function of “randomForest” package of R (Liaw and Wiener, 2002). Prediction accuracy was estimated using a correlation coefficient between phenotypic value and genomic prediction value obtained from validation dataset in 10-fold cross validation. The correlation coefficients from the 10-time replications in 10-fold cross validations were averaged.

### Prediction Accuracies With Selected SNPs

We performed genomic prediction with decreased number of SNPs to estimate the effective SNP numbers for application of GS using low-density markers. Taking into account the positions of SNPs, we examined two types of SNP sampling procedures from the 6,455 mapped SNPs based on the methods described in Cericola et al. (2017). One was the following GWAS-based selection procedure: (i) select the SNP with the highest –log_10_(*P*) of GWAS; (ii) exclude all the SNPs positioned ± *d* from the selected SNP (initial *d* = 10 cM); (iii) if there are still SNPs to select for, move to step *i*; (iv) if all SNPs are excluded or selected, select the SNP which shows the highest -log_10_(*P*) and the distance more than *d = d/*2 from already excluded ones to select; (v) move to step *ii*, and carry out until all SNPs that are located in different positions are selected; (vi) if all SNPs that are located different positions from the selected SNPs, pick up the SNPs in the order of higher values of –log_10_(*P*). The other was the semi-random selection procedure as follows: (i) assign scores (1-6,455) randomly and uniquely for all SNPs; (ii) assuming the assigned scores as –log_10_(*P*), conduct the GWAS-based selection procedure from step *i*. Following this procedure enables a limitation on the inclusion of markers in the same region (Cericola et al., 2017). Numbers of SNPs were set at 10, 25, 50, 100, 250, 500, 1,000, 2,500, 5,000, and 6,455. GWAS was performed by same methods as mentioned above. Using the groups of the selected SNPs, genomic predictions based on GBLUP or RF were carried out, and the prediction accuracies were calculated. For the procedures, 10-fold cross validation with 10-time replications were performed.

## Results

### Genetic Characteristics of the Populations

**Table 1** shows a summary of genetic characteristics of the populations. The average *H*_E_ and *H*_O_ had similar values among the populations, ranging from 0.360 to 0.365 and from 0.356 to 0.358, respectively.

**Table 1:** Summary of population genetics characteristics.

				Based on the mapped SNPs within the same LG			Based on SNPs within the same isotigs		
					
Population	*n*	*H*_E_	*H*_O_	*r*^2^	Ratio of significant SNP pairs (%)		*r*^2^	Ratio of significant SNP pairs (%)	*N*_Q_± SD
N-Kanto	181	0.364	0.356	0.056 (0.050–0.067)	8.6	(7.4–10.0)	0.393	86.8	1.83 ± 0.574
S-Kanto	159	0.365	0.358	0.063 (0.052–0.071)	7.8	(7.2–8.7)	0.394	85.2	1.80 ± 0.565
Kyushu	136	0.360	0.357	0.059 (0.056–0.066)	16.9	(15.6–19.0)	0.395	82.5	1.68 ± 0.611
Total/Mean	476			0.059	11.1		0.394	84.9	1.78 ± 0.585


*The *r*^*2*^ values and ratio of SNP pairs with significant LD per total pairs represent the means of the averages within LGs and isotigs. Values in parentheses represent the range.*

**FIGURE 1:**
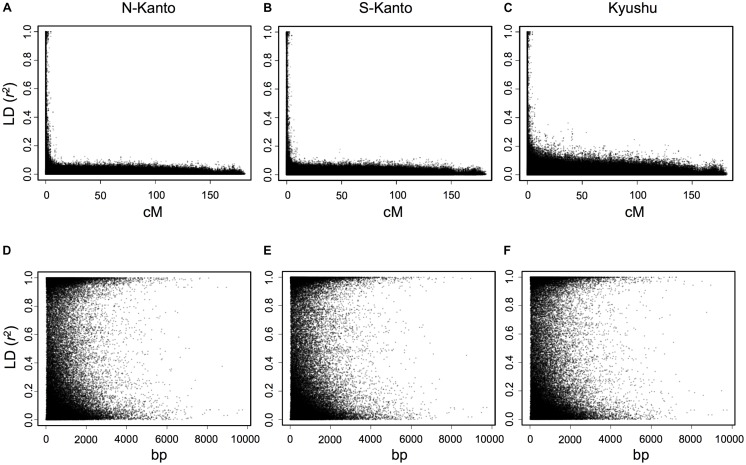
Linkage disequilibrium (*r*^2^) versus map distance (cM) for each population of *C. japonica* plus trees. Upper and lower panels represent LDs within the same LG and within the same isotigs for populations N-Kanto (**A** and **D**, respectively), S-Kanto (**B** and **E**, respectively) and Kyushu (**C** and **F**, respectively). Data derived from the 11 LGs were represented together for each population.

**FIGURE 2:**
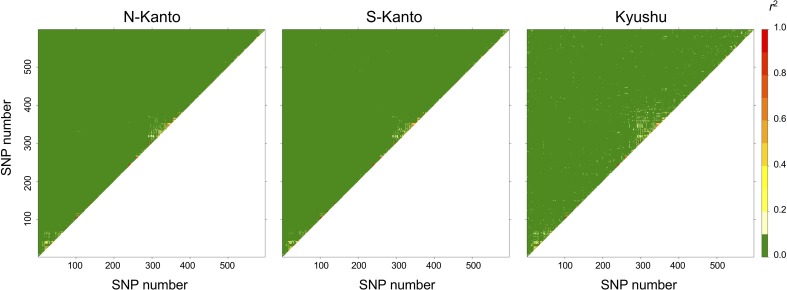
Linkage disequilibrium (LD) between pairs of SNPs as heat map in LG2.

The extent of LDs (*r*^2^) based on the mapped SNPs within the same LGs decayed rapidly within several centimorgans for all populations, and the decay was more rapid in the N-Kanto and S-Kanto populations than in the Kyushu population (**Figures 1A–C**). The average *r*^2^ values showed a similar low level in all populations, ranging from 0.056 to 0.063 (**Table 1**). The average ratio of significant SNP pairs (*p* < 0.01) was higher in Kyushu (16.9%) than in N-Kanto (8.6%) and S-Kanto (7.8%), as shown in **Table 1**. On the other hand, based on SNPs within the same isotigs, which were assembled ESTs, SNP pairs in which LDs extended over 6 kb were observed in all populations (**Figures 1D–F**). The average values of *r*^2^ (0.393–0.395) and the ratio of significant SNP pairs (82.5–86.8) were much higher than those based on the mapped SNPs, but values were similar within the populations (**Table 1**). The distribution of LDs between mapped SNPs within LG2 for each population is shown in **Figure 2**. In LG2, pairs showing higher level of LD (e.g., *r*^2^ > 0.8) were observed only close to the diagonal line in all populations, i.e., SNP pairs located in the vicinity. In the Kyushu population, SNP pairs showing *r*^2^ > 0.1 were more frequently observed relatively far away from the diagonal line than in the N-Kanto and S-Kanto populations. Data for other LGs showed similar trends as was observed in LG2 (**Supplementary Figure S3**).

**FIGURE 3:**
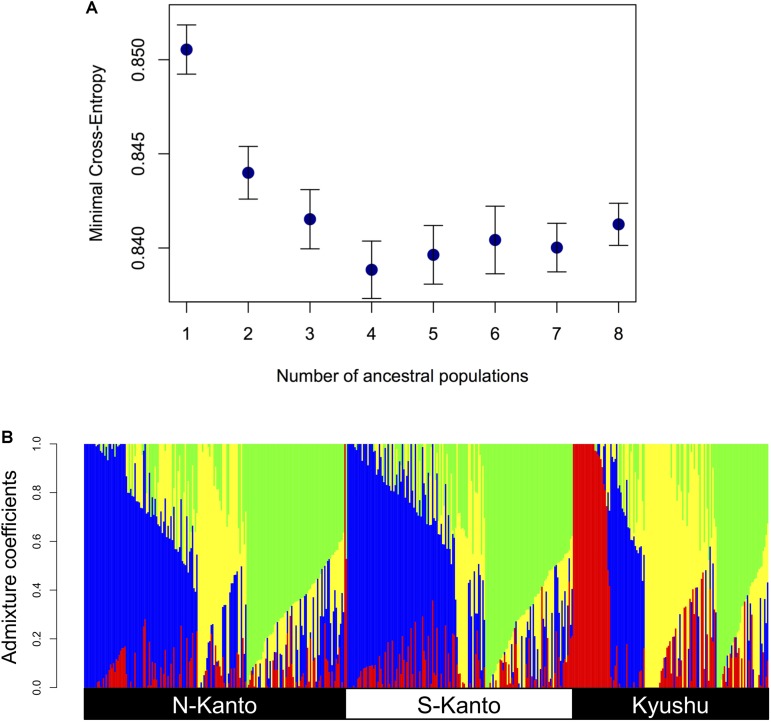
**(A)** Relationship between minimal cross-entropy and number of ancestral populations (*K*) used in ancestry analysis. The values represent the averages of 10 replications. Error bars represent SE. **(B)** Bar-plot of admixture coefficients based on ancestry analysis using the 6,455 mapped SNPs at *K* = 4. Different colors represent different clusters.

**Figure 3** shows the results of ancestry analysis based on the 6,455 mapped SNPs. The cross-entropy criterion minimum occurs at *K* = 4 (**Figure 3A**), and ancestry analysis for the genotypes from the all populations at this value show four clusters in each population but in different proportions (**Figure 3B**). The N-Kanto and S-Kanto populations mainly consisted of three clusters (represented in blue, green and yellow colors). On the other hand, the Kyushu population consisted of clusters represented by yellow, green and red colors, and the blue cluster was a relatively minor one. The mean value of *N*_Q_ for the genotypes in Kyushu was relatively smaller (1.68; **Table 1**) than that in the N-Kanto (1.83) and S-Kanto (1.80) populations.

**FIGURE 4:**
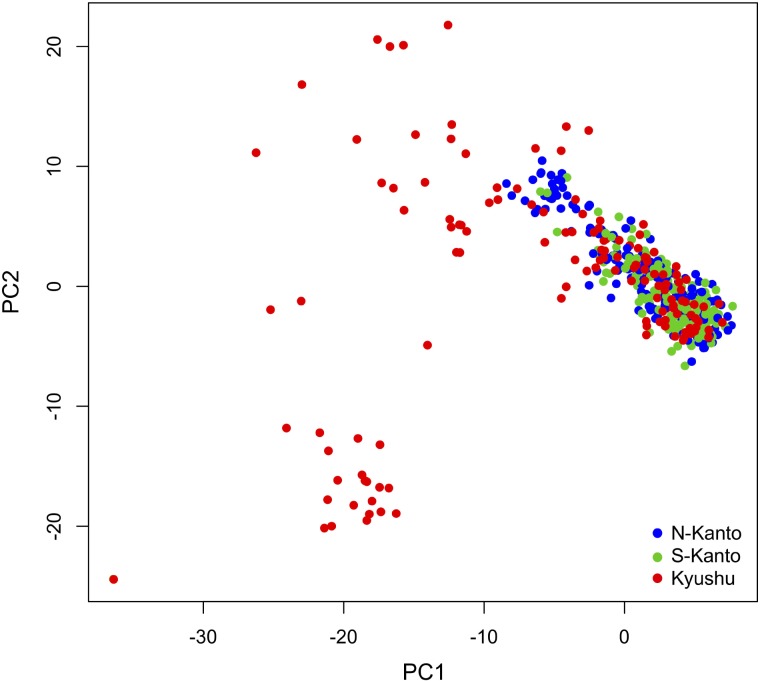
Bi-plot of PCA with PC1 and PC2 based on 6,455 mapped SNPs for N-Kanto, S-Kanto, and Kyushu populations.

**Figure 4** shows a scatter plot of the first and second principal components (PC1 and PC2, respectively) from the PCA result. The genotypes from the N-Kanto and S-Kanto populations showed overlapping and similar distributions. On the other hand, genotypes from the Kyushu population showed a wider ranging distribution with partial overlap in the distributions with those from the two Kanto populations.

### Genome-Wide Association Study

**FIGURE 5:**
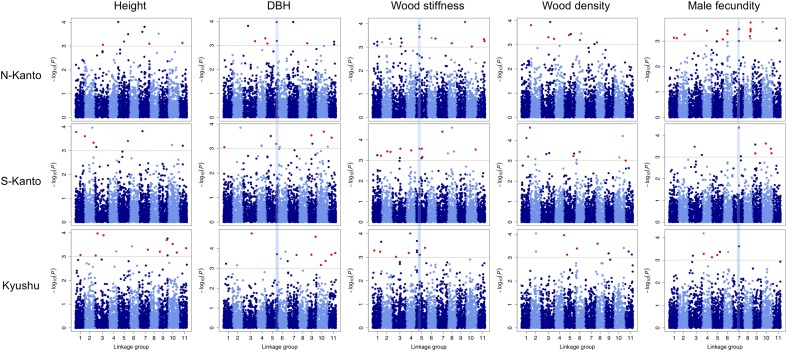
Manhattan plots of GWAS results for the assessed traits in each population. Results for traits of height, DBH, wood stiffness, wood density and male fecundity are shown in columns for the N-Kanto, S-Kanto and Kyushu populations shown in the upper, middle and lower rows, respectively. Vertical blue lines represent the common significant regions shown in **Table 2**. Red circles indicate significant SNPs (– log_10_(*P*) > 3) which were unmapped but estimated their positions based on the LD extent with the mapped ones.

Throughout all traits in the three populations, significant markers in the criterion based on –log_10_(*P*) > 3 were observed in the GWAS results (**Figure 5** and **Supplementary Table S3**). Out of 466 significant SNPs, only two loci that showed FDR < 0.1 were detected (**Supplementary Table S3**); each one SNP was observed for height in the N-Kanto and for wood stiffness in Kyushu populations, respectively. The map positions of the two SNPs were not accurately determined because these were not mapped by Mishima et al. (2018) nor the values of *r*^2^ with paired SNPs were ≤ 0.6 (**Supplementary Table S3**).

**Table 2** shows significant SNPs (–log_10_(*P*) > 3) located close (<10 cM) to the position across the three populations. In total, 13 SNPs were detected, i.e., at ∼131–141 cM in LG5 for DBH, at ∼52–56 cM in LG5 for wood stiffness, and at ∼81–84 cM in LG7 for male fecundity. Particularly for male fecundity, the map position (84.71 cM in LG7) was consistent in the three populations. These loci were matched with *Arabidopsis* homologs by a BLASTN search (**Table 2**).

**Table 2:** Significant SNPs located close (<10 cM) to the position across the three populations identified by GWAS. The positions of SNPs which were not mapped by Mishima et al. (2018) were estimated by the LD calculation with the mapped SNPs, and the paired SNPs and *r*^2^ were described.

Trait	SNP	Isotig	Position in isotig (bp)	LG	Position (cM)	Population	–log_10_(*P*)	Paired SNP	LD (*r*^2^)	Description	E-value	*Arabidopsis* homolog locus
DBH	AX-115733559	reCj33053	208	5	131.911	S-Kanto	3.197	AX-115733531	0.9994	Disease resistance-responsive (dirigent-like protein) family protein	1.4E-33	AT5G42500.1
	AX-115702430	reCj23615	194	5	134.464	N-Kanto	3.977			UDP-N-acetylglucosamine (UAA) transporter family	3.0E-155	AT4G31600.1
	AX-115712388	reCj21983	1258	5	138.325	N-Kanto	3.191			alpha/beta-Hydrolases superfamily protein	1.6E-57	AT3G48090.2
	AX-115717873	reCj12784	2252	5	140.858	Kyushu	3.712			ENTH/ANTH/VHS superfamily protein	0.0E+00	AT5G35200.1
Wood stiffness	AX-116812637	reCj18655	2193	5	52.117	N-Kanto	3.916			Transducin/WD40 repeat-like superfamily protein	1.7E-148	AT4G35560.1
	AX-115676628	reCj16814	464	5	52.117	N-Kanto	3.787			Expressed protein	1.4E-79	AT5G66005.3
	AX-115674887	reCj18655	684	5	52.117	N-Kanto	3.432	AX-116812637	0.9923	Transducin/WD40 repeat-like superfamily protein	1.7E-148	AT4G35560.1
	AX-115734315	reCj17876	4099	5	52.117	S-Kanto	3.565	AX-115734283	0.9995	With no lysine (K) kinase6	5.7E-172	AT3G18750.3
	AX-115684475	reCj11756	1820	5	56.303	Kyushu	3.100			Unknown protein	9.8E-141	AT2G25270.1
Male fecundity	AX-115677828	Cj.5263_1	463	7	81.948	N-Kanto	3.001			Co-factor for nitrate, reductase and xanthine dehydrogenase 5	7.0E-07	AT5G55130.1
	AX-115722963	reCj21237	876	7	84.710	N-Kanto	3.471			RING/U-box protein	3.7E-35	AT3G05670.1
	AX-115723152	reCj18165	2314	7	84.710	S-Kanto	4.365	AX-115700261	0.9994	Formin homology 1	2.1E-153	AT3G25500.1
	AX-115695381	reCj19723	427	7	84.710	Kyushu	3.617			Octicosapeptide/Phox/Bem1p (PB1) domain-containing protein/tetratricopeptide repeat (TPR)-containing protein	2.3E-139	AT2G25290.3


### Prediction Accuracies Based on the Three Models

We compared genomic prediction accuracies among the three models, GBLUP, BayesB and RF (**Table 3**). For height, RF showed the highest prediction accuracies in N-Kanto (0.267) and Kyushu (0.468), whereas in S-Kanto the highest accuracy was obtained with BayesB and the value was close to zero (–0.026). For DBH, similar but slightly better results were observed compared to those for height; RF showed the highest accuracies in N-Kanto (0.299) and Kyushu (0.523), but in S-Kanto, BayesB again showed the highest accuracy but the value was close to zero (0.033). For wood stiffness, GBLUP and RF showed higher accuracies in S-Kanto (0.313) and Kyushu (0.531), respectively; in N-Kanto, the highest accuracy was obtained with BayesB and the value was close to zero (–0.099). In terms of wood density, GBLUP and RF showed higher accuracies in N-Kanto (0.247) and Kyushu (0.193), respectively; in S-Kanto, the highest accuracy was obtained for RF but the value was close to zero (–0.076). For male fecundity, GBLUP had higher accuracies for all populations (N-Kanto, 0.617; S-Kanto, 0.357; Kyushu, 0.634).

**Table 3:** Mean (±SE) accuracies based on all 32,036 SNPs.

Trait	Model	N-Kanto	S-Kanto	Kyushu
Height	GBLUP	0.120 ± 0.012	-0.040 ± 0.013	0.446 ± 0.006
	BayesB	0.114 ± 0.016	**-0.026 ± 0.021**	0.432 ± 0.006
	RF	**0.267 ± 0.017**	-0.089 ± 0.037	**0.468 ± 0.007**
DBH	GBLUP	0.253 ± 0.006	-0.001 ± 0.016	0.512 ± 0.006
	BayesB	0.209 ± 0.010	**0.033 ± 0.020**	0.505 ± 0.005
	RF	**0.299 ± 0.019**	-0.052 ± 0.030	**0.523 ± 0.005**
Wood stiffness	GBLUP	-0.140 ± 0.018	**0.313 ± 0.010**	0.520 ± 0.006
	BayesB	**-0.099 ± 0.013**	0.216 ± 0.012	0.500 ± 0.005
	RF	-0.124 ± 0.021	0.088 ± 0.031	**0.531 ± 0.008**
Wood density	GBLUP	**0.247 ± 0.009**	-0.182 ± 0.016	0.082 ± 0.019
	BayesB	0.193 ± 0.013	-0.118 ± 0.015	0.129 ± 0.011
	RF	0.144 ± 0.017	**-0.076 ± 0.018**	**0.193 ± 0.014**
Male fecundity	GBLUP	**0.617 ± 0.004**	**0.357 ± 0.008**	**0.634 ± 0.003**
	BayesB	0.609 ± 0.003	0.322 ± 0.009	0.616 ± 0.003
	RF	0.548 ± 0.006	0.300 ± 0.030	0.629 ± 0.005


*Highest value in each trait and population combination is in bold.*

Although the best models were different for the different populations and traits, GBLUP and RF were overall better models than BayesB. Among traits, male fecundity was the most predictable, and height and wood density showed lower accuracies. Based on the accuracies of the best model, the Kyushu population showed the best predictability for all traits except for wood density. Between the two Kanto populations, accuracies of almost all traits were higher in the N-Kanto population than in the S-Kanto population, except for wood density. Therefore, the prediction accuracies were in descending order Kyushu, N-Kanto and S-Kanto.

### Prediction Using Selected SNPs

**FIGURE 6:**
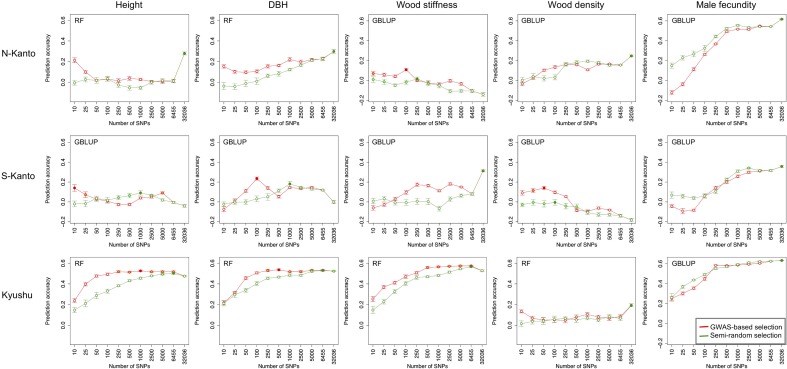
Transitions of prediction accuracies as a function of the number of SNPs selected, based on the GWAS result for each population–trait combination. Results for traits of height, DBH, wood stiffness, wood density and male fecundity are shown in columns for the N-Kanto, S-Kanto and Kyushu populations shown in the upper, middle and lower rows, respectively. Orange and light-green colored circles indicate that those are not significantly different from the highest values (red and green colored circles, respectively) with *t*-test (*p* ≥ 0.05). Error bars represent SE. The model (G-BLUP or RF) showing better prediction accuracies in **Table 3** was used.

**Figure 6** shows transitions of prediction accuracies with GBLUP or RF (higher accuracy model selected) as a function of the number of SNPs selected by GWAS-based selection or semi-random selection procedures. For all traits, except for male fecundity, and all populations, prediction accuracies with SNPs of GWAS-based selection were higher than with semi-random selection. Based on results of the GWAS-based selection, high accuracies that were not significantly different than the highest ones (*p* > 0.05) were observed with relatively small numbers of SNPs (≤500) for height in S-Kanto and Kyushu, DBH in S-Kanto and Kyushu, wood stiffness in N-Kanto and Kyushu, and wood density in S-Kanto. For male fecundity, prediction accuracies with a small number of SNPs in the semi-random selection were higher than for GWAS-based selection, and similar transitions of accuracies with larger samplings of SNPs (≥500) were observed for both selection procedures in all populations. Particularly in the S-Kanto population, high accuracy using 2,500 SNPs selected by the semi-random selection procedure was observed for male fecundity. Prediction accuracies for height and DBH in N-Kanto, wood stiffness in S-Kanto, wood density in N-Kanto and Kyushu, and male fecundity in N-Kanto and Kyushu were significantly higher at all SNPs (32,036) than that at the other sampling levels.

## Discussion

### Genetic Characteristics of the Populations

For the first-generation plus tree populations of *C. japonica*, LDs at the LG level rapidly decayed in all populations; however, LDs in the Kyushu population were slightly higher than that in the others. As Uchiyama et al. (2014) mentioned, domestication and breeding programs for Japanese cedar are still in their infancy and the plus trees have not suffered from diversity losses caused by a domestication bottleneck. On the other hand, LDs at the isotig level showed high values at a distance of over 6 kb. Moritsuka et al. (2012) reported that LD was extensive and did not decay even at a distance of 100 kb in non-coding regions of the genome of this species. Based on the previous findings, it is considered that longer LDs at the genomic DNA level remain than were observed in this study for *C. japonica* plus trees because the isotigs consisted of coding regions.

The results of the genetic structure based on ancestral analysis showed that individuals in the Kyushu population represented a less effective number of clusters per individual (*N*_Q_) than in the other two populations. Additionally, the genotypes from the Kyushu population showed a wider range distribution than did those from the two Kanto populations in the PCA. From these results, characteristics in the Kyushu population were different from the other populations, in which the distribution of weak but positive LDs throughout the chromosomes, smaller *N*_Q_ and higher genetic diversity were observed. On the other hand, differences between the two Kanto populations were slight compared to the differences between the Kyushu population and the two Kanto populations.

Population histories reflect the genetic characteristics in human and several animal and crop species (Dunning et al., 2000; Meadows et al., 2008; Slatkin, 2008; Gray et al., 2009; Rossi et al., 2009; Carneiro et al., 2011; Hamblin et al., 2011; Akagi et al., 2016; Campoy et al., 2016). Genetic characteristics of *C. japonica* plus trees are considered to be based on the common genetic background of natural populations (Tomaru, 1992; Miyamoto et al., 2014). According to Tsukada (1982), *C. japonica* had multiple refugia in Japan during the last glacial period where relatively moist, cool climates prevailed. Its expansion began from scattered full glacial centers of distribution ∼15,000 years ago, reaching its maximum abundance from 7,000 to 2,000 years ago (Tsukada, 1982). From more than 10,000 years ago, *C. japonica* expanded from refugias, such as the Izu Peninsula along the Pacific coast or Wakasa Bay along the Japan Sea coast (Tsukada, 1982). Additionally, based on phylogeography and species distribution modeling, a “cryptic” refugia was thought to be present in northern Tohoku (Kimura et al., 2014). The current genetic structure of natural forests of *C. japonica* is considered to reflect the locations of refugia because offspring of the survivors would have colonized out from refugia during the interglacial period, and genetic differentiation between the isolated populations and other populations is likely to have increased during their isolation (Tsumura et al., 2014). The early stages of forestation of *C. japonica* started more than 500 years ago (Tokugawa, 1974). The genetic structure of current artificial forests and plus-tree populations of the species were considered to be reflected the geography of the natural populations as shown in Uchiyama et al. (2014).

In this study, almost similar LD and population structures were observed between the two Kanto populations. This suggests that *C. japonica* individuals in the Kanto region originating from refugia along the Pacific coastal area, including plus trees in both of the Kanto populations. On the other hand, on Kyushu Island, *C. japonica* became extinct before 25,000 years ago, and the initial stock of this species was likely brought from Honshu Island by prehistoric man ∼2,500 years ago (Tsukada, 1982). In this study, the Kyushu population showed higher LDs and higher diversity based on PCA than those of other populations. A similar LD result was also reported by Uchiyama et al. (2014) in which a subpopulation from the Kyushu region exhibited the highest level of LD. As Uchiyama et al. (2014) discussed, population history and forestry management have led to higher LD. Higher LD observed in the Kyushu population might reflect founder populations that have recently expanded from relatively small sizes (Shifman and Darvasi, 2001). The higher diversity of the Kyushu population suggests the existence of multiple founders from different genetic backgrounds. Additionally, clonal forestry using cutting propagation has been traditionally conducted in Kyushu since 500 years ago (Miyajima, 1989) while planting seedling has been the main strategy in the Kanto region. The prevailing method of vegetative propagation in Kyushu makes recombination unlikely to occur and may also maintain LD (Barnaud et al., 2006; Uchiyama et al., 2014; Minamikawa et al., 2017). The result that mean *N*_Q_ value based on ancestry analysis was smaller in the Kyushu population than in the others means that admixture among individuals occurred with less frequency due to the forestry characteristics in the area. These differences in terms of population histories and forestry regimes affect the genetic characteristics of *C. japonica* plus tree populations.

### Detection of Significant Genome Regions by GWAS

Among the results of GWAS for the five traits in the three populations, the only two SNP loci that showed FDR < 0.1 were detected for height in the N-Kanto and for wood stiffness in the Kyushu population. From the result that such few loci were significant based on the FDR criterion, the detection power of GWAS seems to not be high in this study. Such low GWAS power can be attributed to using unrelated first-generation plus trees as assessment populations. Minamikawa et al. (2017) observed that GWAS detection power in citrus was higher using parental and F_1_ populations than in the parental population only. In this study, using only the unrelated first-generation plus tree genotypes probably caused the low detection power of GWAS.

Even though the detectability of GWAS was low, the common significant genomic regions throughout the three populations were detected by performing GWAS separately for the traits of DBH, wood stiffness and male fecundity. Generally in GWAS, there is potential to detect false associations between markers and traits where no causal relation exists (Platt et al., 2010). On the other hand, GWAS using multiple different populations is considered to compensate for such low detection power and to be useful for avoiding detection of such false associations. Additionally, the low LD observed in *C. japonica* should reduce the occurrence of spurious associations (Abdurakhmonov and Abdukarimov, 2008; Hamblin et al., 2011). Thus, the common significant genomic regions detected by GWAS using all populations might be expected to be associated with loci with large effects contributing to the phenotype of corresponding traits of *C. japonica*, particularly male fecundity. It is possible that the significant loci detected by GWAS in this study were located close to causative genes. Thus, GWAS results such as those obtained in this study could provide important insights in future genetic research on the traits of this species.

### Difference of Accuracies of Genomic Prediction Depend on the Traits, Models and Populations

For genomic prediction accuracy, trait heritability is one of the important factors as described in previous studies (Hayes and Goddard, 2010; Grattapaglia and Resende, 2011). The ranges of broad-sense heritability which were previously reported for the traits assessed in this study were as follows: 0.37–0.72 for height (Tamura et al., 2006; Fukatsu et al., 2011), 0.21–0.52 for DBH (Fujisawa et al., 1994; Tamura et al., 2006; Fukatsu et al., 2011), 0.65 for wood stiffness (Fujisawa et al., 1994), 0.78–0.88 for wood density (Tamura et al., 2006; Fukatsu et al., 2011), and 0.94 for male fecundity (Nakamura, 2015). In this study, prediction accuracies for male fecundity were the highest, and those for wood density were the lowest among the assessed traits. Therefore, although there was not always positive relationship between the broad-sense heritability and prediction accuracies, the both variables for male fecundity were higher than that for the other traits.

The differences in the genetic architecture between traits could be expected to affect the relative efficacy of different prediction methods (Spindel et al., 2015). In this study, the prediction accuracies with GBLUP were higher than those obtained with BayesB for almost all assessed traits, except for traits with accuracies that were close to zero. The BayesB method is regarded as useful only if markers pick up strong associations with QTL (Jannink et al., 2010). Thus, the low performance of BayesB observed in this study suggests that the assessed traits were controlled by many QTLs in *C. japonica*. The accuracies obtained by RF also showed high values for prediction of growth and wood stiffness in this study. When GS is applied for selection from clonal populations, prediction of dominance effects and the effect of epistatic interactions of specific allelic patterns at several loci might be important for predicting total genetic values, and such effects would have to be added to the model (Grattapaglia and Resende, 2011). Since a non-linear prediction model such as RF may be particularly useful when the relationships between predictors and responses are non-linear, as would occur if epistatic effects account for a significant portion of genetic variation of a target trait (Jannink et al., 2010). Non-parametric regression methods that may also account for non-additive effects have also been proposed (Gianola and van Kaam, 2008; González-Recio et al., 2008; Bennewitz et al., 2009; Neves et al., 2012). Of these, it is possible that traits such as growth or wood properties of *C. japonica* plus tree genotypes would be influenced by non-additive genetic factors.

We also observed accuracy differences among populations; the prediction accuracies in the Kyushu population were generally the highest, followed in order by those in the N-Kanto and S-Kanto populations. Genomic prediction accuracy could be attributed to two main factors: (1) prediction based on LD between markers and QTL; (2) prediction based on genomic relationships arising from population structure (Daetwyler et al., 2012). In *C. japonica* plus trees, although results depend on traits and populations, we confirmed moderate accuracies (0.5–0.6) for some traits in the Kyushu population and for male fecundity in the N-Kanto population. Among the populations, the Kyushu population showed slightly higher LD, corresponding to the order of prediction accuracies. The extent of LD is one of the key factors for genomic prediction because it is based on the LD between markers and causal QTLs (Heffiner et al., 2009; Jannink et al., 2010; Brachi et al., 2011; Korte and Farlow, 2013). Additionally, in the Kyushu population, relatively high genetic diversity and a different ancestral (red colored) cluster were observed compared with that in the others. Our results suggest that such differences in genetic characteristics among the populations, including both of LD and genetic structure, reflected the prediction accuracies.

### Future Application of Genomic Prediction in *C. japonica*author Improvement

In terms of successfully applying GS models, moderate to high prediction accuracy is required. In this study, we observed moderate prediction accuracies for DBH and wood stiffness in the Kyushu population and male fecundity in the N-Kanto and Kyushu populations. Further, for general applications of GS, prediction of breeding values for individuals from the same population but not particularly closely related to the training individuals, or ‘unrelated’ individuals, is required (Meuwissen, 2009) and are used in the most promising applications of GS (Wray et al., 2007; Meuwissen, 2009). Furthermore, in the prediction of genomic estimated breeding value, the phenotype of individuals is regressed against genetic markers in the training population. The ideal phenotype would be true breeding values (TBV) measured in a population of unrelated individuals without selection (Garrick et al., 2009). In this study, we constructed genomic prediction models using multiple populations consisting of unrelated plus trees of *C. japonica* based on clonal abilities, which would be assumed as the TBV of genotypes. Therefore, the prediction models constructed in this study could be generally considered to be applicable to other individuals within the same population, although dependent on traits or populations. Namely, the models would allow to predict the traits of other first-generation plus trees which had not been phenotyped and to select additional superior trees from natural or artificial forests. By phenotyping such selected individuals in future, it would be possible to verify the effectiveness of the prediction models for general GS applications.

In GS application, high genotyping cost would be another problem. Reducing the SNP number is an effective way to cut genotyping cost. Pre-selecting SNPs could be crucial for improving the quality of genomic predictions (Croiseau et al., 2011). In this study, we examined two procedures for SNP selection, i.e., GWAS-based selection and semi-random selection. Our results showed that the former was more effective for traits of growth and wood properties, and similar accuracies were obtained by both selection procedures with large size (≥500) SNPs for male fecundity. Cericola et al. (2017) used selection procedures similar to those in the present study and also showed that GWAS-based SNP selection increased the accuracy compared to that for semi-random selection. It is suggested that prediction accuracies can be improved by SNP selection based on the GWAS result (Spindel et al., 2015, 2016). Also in this study, GWAS-based selection may be a common and effective way to predict assessed traits, although prediction accuracies would be decreased for some traits and populations. From the results of the GWAS-based selection, there were differences in the optimum SNP number for genomic prediction depending on traits and populations. Such differences in transitioning patterns might be attributable to differences in genetic architecture between traits or populations. Nevertheless, for populations which showed low prediction accuracies, additional SNPs development might be necessary to improve accuracy of genomic prediction in *C. japonica*. Advanced genome sequencing of the species would enable us to develop more high-density SNPs in the near future.

Since the progress of *C. japonica* breeding (currently in the second generation) is relatively delayed compared with other coniferous species, such as *Pinus taeda* (in the third generation; (North Carolina State University Cooperative Tree Improvement Program [NCSUCTIP], 2017) and *Pinus radiata* (in the third generation; Dungey et al., 2009). Currently, forward selection of the second-generation plus trees has been undertaken, and evaluations of their offspring and clones have been started at clonal or progeny test sites for *C. japonica*. Genome-wide studies might have potential to accelerate the breeding cycle of this species. In a simulation study of GS for *C. japonica*, although prediction accuracies of models constructed in an early generation decreased with each generation, genetic gains were maintained for several generations (Iwata et al., 2011). Therefore, it is expected that the models constructed for the first generation would be also useful for predictions in several subsequent generations. In several empirical studies for other coniferous species, high accuracies (>0.7) has been reported for consideration of full-sib families (Resende et al., 2012a,b; Beaulieu et al., 2014b; Lenz et al., 2017). These findings suggest that under certain conditions of high relatedness, long-range LD is likely a more potent factor of accuracy than short-range LD (Grattapaglia and Resende, 2011; Resende et al., 2012a; Beaulieu et al., 2014b). In this case, prediction accuracies obtained in the promoted generation would be expected to increase from the basic ones observed in this study using the first-generation plus trees. Accuracies in the next generation will be examined in future studies in *C. japonica*.

## Conclusion

This is the first empirical study of both GWAS and genomic prediction in *C. japonica* using multiple populations of unrelated first-generation plus tree genotypes. Among the assessed populations, the genetic characteristics including the extent of LD and genetic structure were different, considering that such characteristics are tied to population histories. We demonstrated the possibility of detection significantly contributing to genomic regions for traits by GWAS using multiple populations. In addition, we showed the basic potential of genomic prediction and empirically revealed the genetic characteristics as determinative factors of prediction accuracy using multiple populations. Furthermore, we demonstrated an effective strategy of SNP selection for genomic prediction using the GWAS results. Our present study suggests the potential of GWAS and GS for improvement of the species.

## Author Contributions

YH and AW conceptualized the present research. EF, KM, TI, MTs, and MK did phenotyping. TH generated genotyping data. YH, EF, KM, KT, and MiTa conducted data analysis. MaT provided technical assistance with data analysis. YH wrote the manuscript.

## Conflict of Interest Statement

The authors declare that the research was conducted in the absence of any commercial or financial relationships that could be construed as a potential conflict of interest.
