# Supplementary material for: Potential of Genome-Wide Studies in Unrelated Plus Trees of a Coniferous Species, Cryptomeria japonica (Japanese Cedar)
Source: Front Plant Sci. 2018 Sep 10;9:1322. doi: 10.3389/fpls.2018.01322 (PMC6141754; doi:10.3389/fpls.2018.01322)
Supplement: Supplementary file 4 [file Presentation_1.pdf]

Supplementary Material

Potential of genome-wide studies in unrelated plus trees of a coniferous species, *Cryptomeria japonica* (Japanese cedar)

Yuichiro Hiraoka*, Eitaro Fukatsu, Kentaro Mishima, Tomonori Hirao, Kosuke M. Teshima, Miho Tamura, Miyoko Tsubomura, Taiichi Iki, Manabu Kurita, Makoto Takahashi, Atsushi Watanabe

* **Correspondence:** Corresponding Author:yhiraoka@affrc.go.jp

1.1 Supplementary Figures

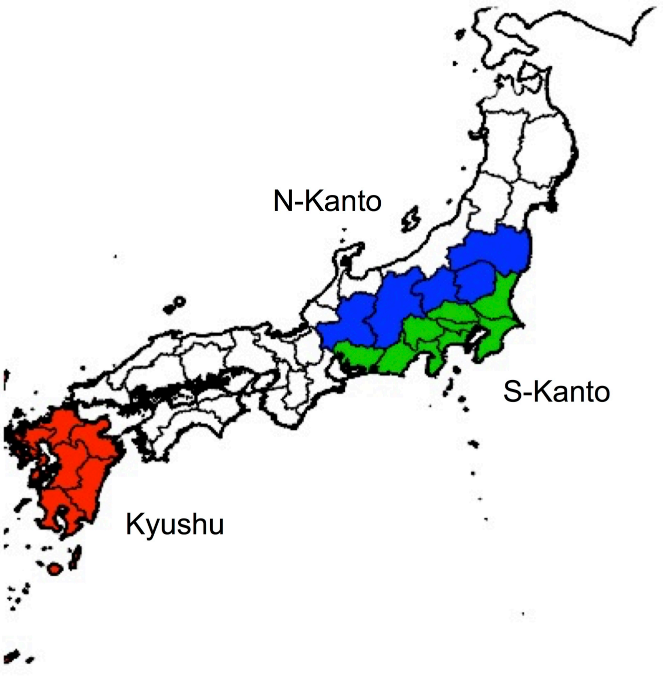

Supplementary Figure 1. Locations of breeding area.

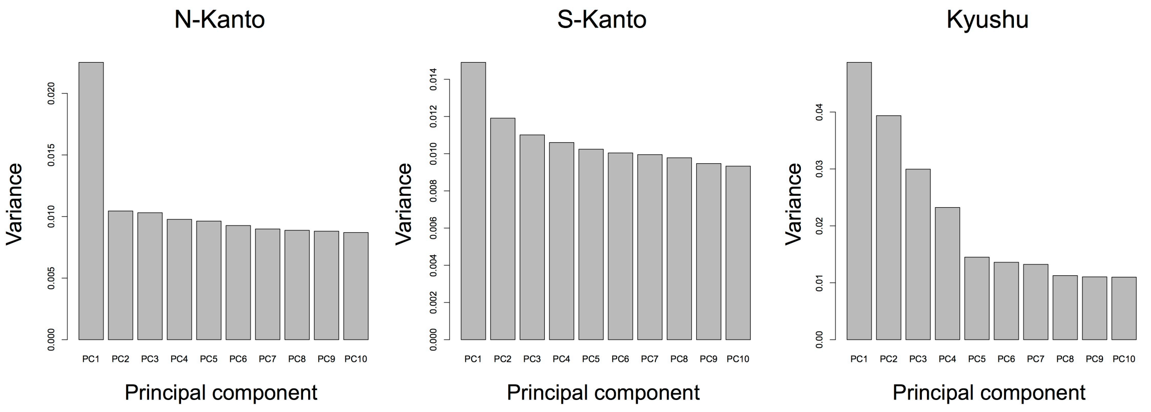

Supplementary Figure 2. Variances of principal component scores for the assessed populations.

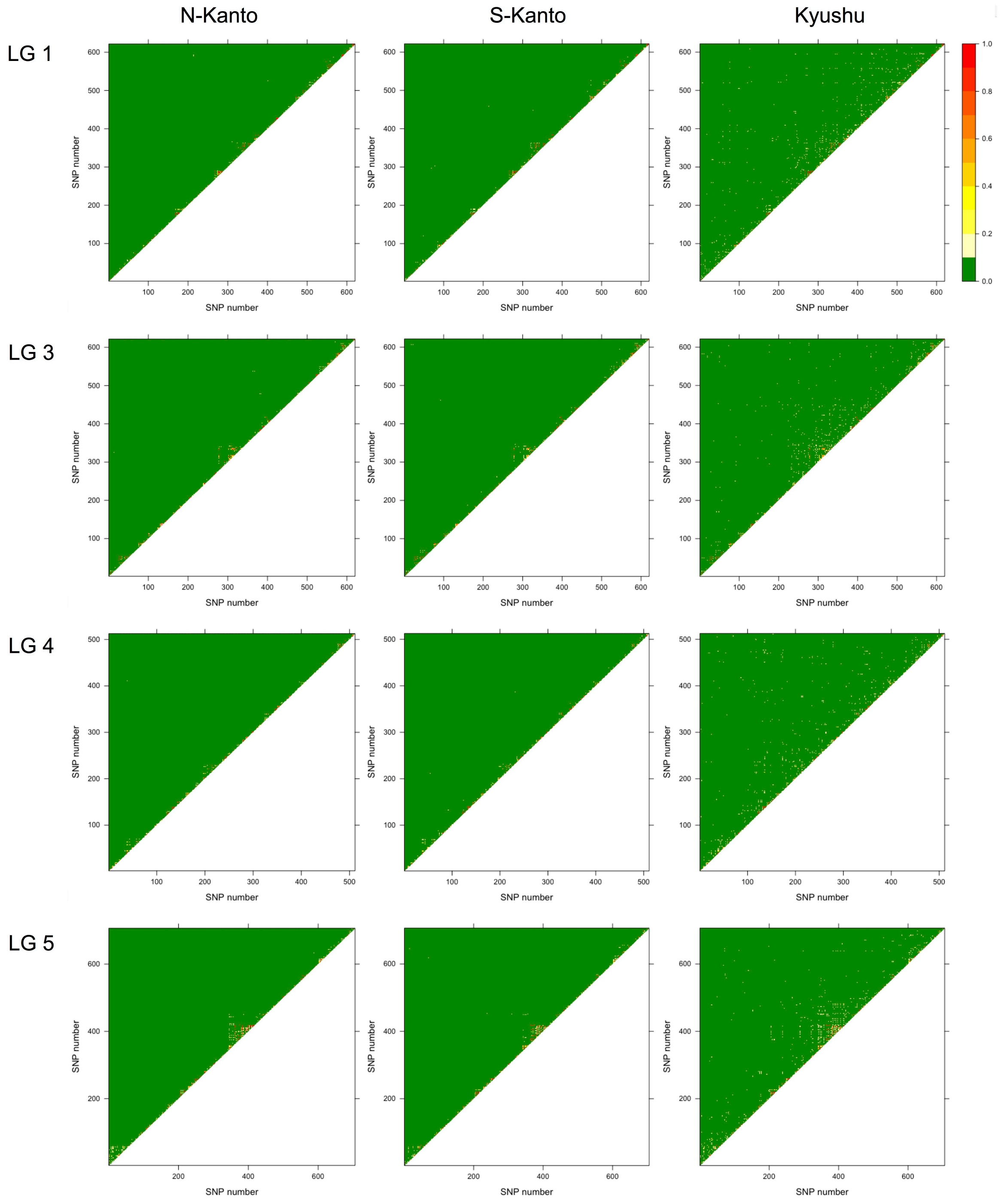

Supplementary Figure 3. LD between pairs of SNPs as heat map within the linkage groups.

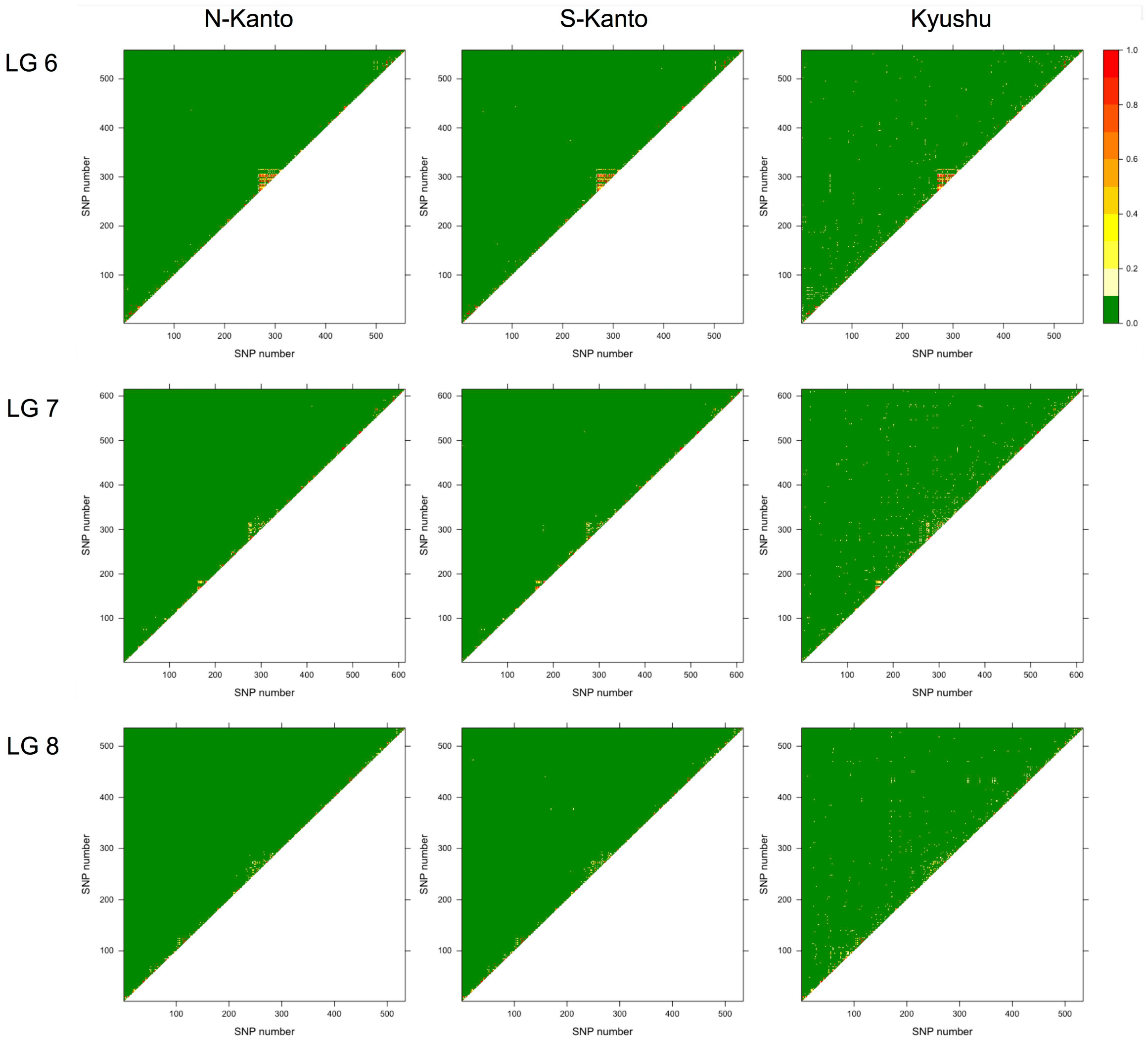

Supplementary Figure 3. Continued

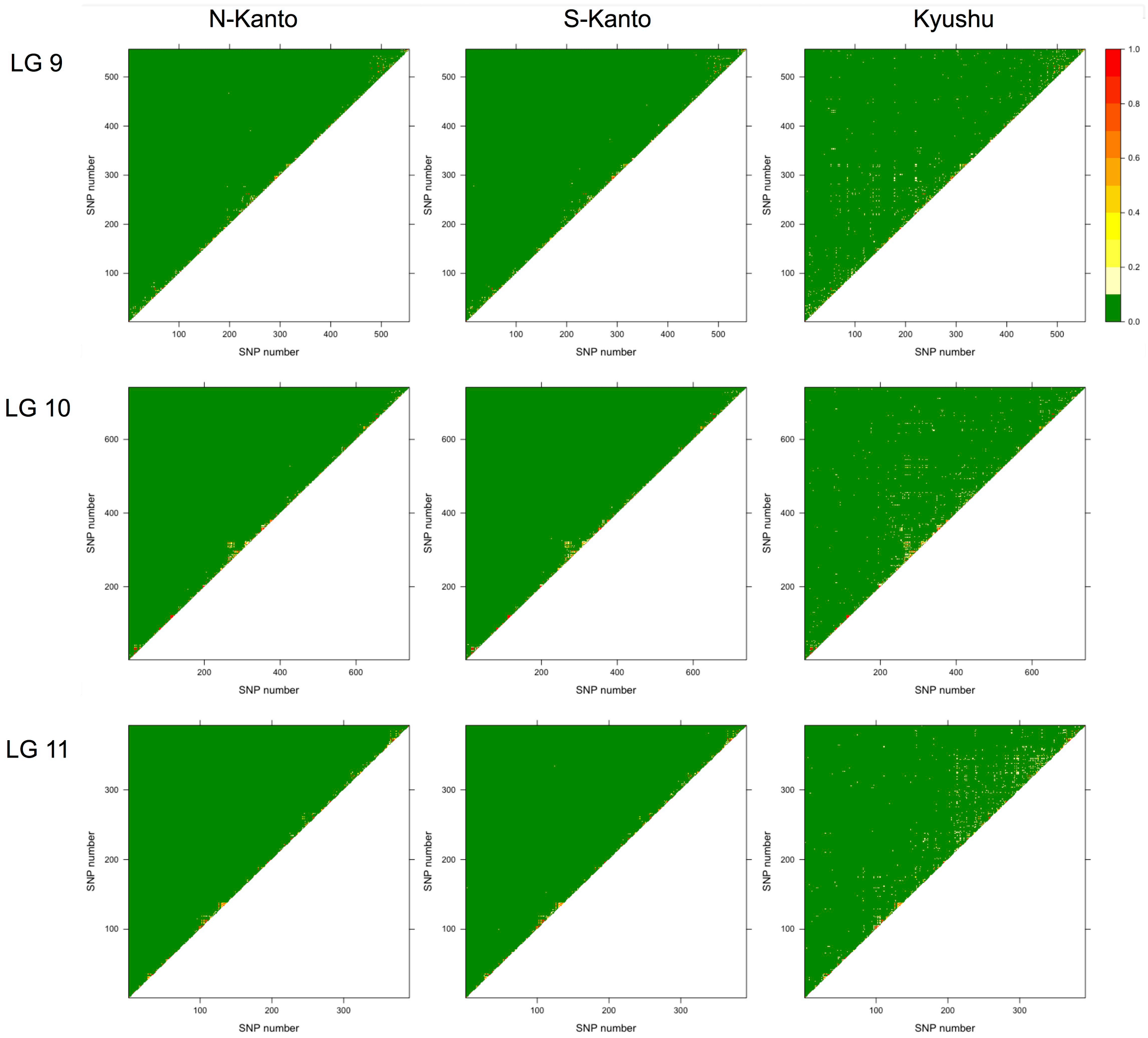

Supplementary Figure 3. Continued
